# Lesion assessment in multiple sclerosis: a comparison between synthetic and conventional fluid-attenuated inversion recovery imaging

**DOI:** 10.3389/fneur.2025.1537465

**Published:** 2025-03-12

**Authors:** Roald Ruwen Essel, Britta Krieger, Barbara Bellenberg, Dajana Müller, Theodoros Ladopoulos, Ralf Gold, Ruth Schneider, Carsten Lukas

**Affiliations:** ^1^Institute of Neuroradiology, St. Josef Hospital Bochum, Ruhr-Universität Bochum, Bochum, Germany; ^2^Department of Neurology, St. Josef Hospital Bochum, Ruhr-Universität Bochum, Bochum, Germany

**Keywords:** multiple sclerosis, lesion segmentation, synthetic MRI, FLAIR imaging, volumetry, MDME

## Abstract

**Background and purpose:**

Magnetic resonance imaging (MRI)-based lesion quantification is essential for the diagnosis of and prognosis in multiple sclerosis (MS). This study compares an established software's performance for automated volumetric and numerical segmentation of MS brain lesions using synthetic T2-weighted fluid-attenuated inversion recovery (FLAIR) MRI, based on a multi-dynamic, multi-echo sequence (MDME), vs. conventional FLAIR imaging.

**Methods:**

To ensure comparability, 3D FLAIR images were resampled to 4 mm axial slices to match the synthetic images' slice thickness. Lesion segmentation was performed using the Lesion Prediction Algorithm within the Lesion Segmentation Toolbox. For the assessment of spatial differences between lesion segmentations from both sequences, all lesion masks were registered to a brain template in the standard space. Spatial agreement between the two sequences was evaluated by calculating Sørensen–Dice coefficients (SDC) of the segmented and registered lesion masks. Additionally, average lesion masks for both synthetic and conventional FLAIR were created and displayed as overlays on a brain template to visualize segmentation differences.

**Results:**

Both total lesion volume (TLV) and total lesion number (TLN) were significantly higher for synthetic MRI (11.0 ± 12.8 mL, 19.5 ± 12.1 lesions) than for conventional images (6.1 ± 8.5 mL, 17.9 ± 12.5 lesions). Bland–Altman plot analysis showed minimal TLV differences between synthetic and conventional FLAIR in patients with low overall lesion loads. The intraclass coefficient (ICC) indicated excellent agreement between both measurements, with values of 0.88 for TLV and 0.89 for TLN. The mean SDC was 0.47 ± 0.15.

**Conclusion:**

Despite some limitations, synthetic FLAIR imaging holds promise as an alternative to conventional FLAIR for assessing MS lesions, especially in patients with low lesion load. However, further refinement is needed to reduce unwanted artifacts that may affect image quality.

## 1 Introduction

Multiple sclerosis (MS) is currently the most common chronic, inflammatory immune-mediated disease of the central nervous system with an increasing incidence each year (1, 2). Demyelination and axonal damage are the hallmarks of MS and are responsible for disseminated neurological deficits (3). Magnetic resonance imaging (MRI) plays a key role in the primary diagnosis as well as in decision-making during the clinical course of treatment. MRI provides detailed information that supports diagnosis, classification, and ongoing treatment monitoring of MS (4). According to the latest McDonald criteria, identification and quantification of focal lesions in the brain and spinal cord are essential for the diagnosis of MS (5, 6). Therefore, the number and characteristics of these lesions play a crucial role in treatment. Lesions located at the juxtacortical or periventricular regions are typical for MS (7). T2-weighted fluid-attenuated inversion recovery (FLAIR) sequences are highly sensitive for detecting and quantifying MS-related lesions and are an integral part of the latest consensus recommendations for the use of MRI in MS (8). However, balancing the need for precise, standardized MRI protocols with the time constraints of routine clinical practice remains a challenge. In this context, synthetic MRI techniques which enable the generation of multiple image contrasts from a single sequence, are of particular interest. The multi-dynamic, multi-echo (MDME) sequence (also referred to as Synthetic MRI) captures multiple MR-contrasts in one time-efficient acquisition. This approach allows for a standardized protocol while significantly reducing scan times (9). In combination with the corresponding postprocessing software (SyMRI^®^), which can be integrated into clinical PACS (picture archiving and communication system), this quantitative imaging sequence generates (perfectly) registered T1, T2 PD, and FLAIR-weighted images and additionally provides the possibility of modulating different sequence timing parameters (including synthetic TR, TE, and TI) to generate pathology-specific image contrasts. Thus, MDME can shorten total scan time by consolidating multiple contrast-weighted sequences into a single acquisition. Furthermore, MDME technology enables greater standardization across different radiological facilities by using predetermined/fixed sequence parameters which enhances consistency and uniformity in radiological assessments. Additionally, the MDME-sequence can be used to generate a variety of quantitative parameters, namely volumes and segmentation maps of total brain, gray and white matter, and advanced quantitative measures such as maps of myelin, proton density and T1 and T2 relaxation times, which are beyond the focus of this study (10). As a trade-off in comparison to conventional high-resolution 3D-MRI the current resolution of the MDME sequence is 2D-anisotropic with an in-plane pixel size 1 mm × 1 mm and 4 mm slice thickness.

Limited data suggest that the quality of synthetic images is comparable to conventionally acquired images, particularly for synthetic T1 and T2 contrasts, although larger datasets are needed to confirm these findings (11, 12).

In this study, we evaluated the quality of MS-lesion assessment using synthetic FLAIR images on a representative large cohort of MS patients in clinical practice and compared it to conventional FLAIR imaging. We thereby created the perspective of a significant reduction of acquisition time by capturing all essential key sequence modalities in one scan while also offering an improvement in standardization and comparability. Hence, we hypothesize that synthetic FLAIR sequences are a viable choice for evaluating MS lesions.

## 2 Materials and methods

### 2.1 Study participants

In this retrospective study, patients with a confirmed diagnosis of MS according to the 2017 McDonald criteria and aged between 18 and 78 years were enrolled. Inclusion criteria were further based on the availability of a standardized in-house MRI examination, including synthetic MRI. Exclusion criteria were poor image quality or missing FLAIR data and pre-existent comorbidities that could interfere with the study assessments, such as microangiopathy, ischemic or hemorrhagic strokes (acute and past incidents), progressive multifocal encephalopathy, cerebral tumors, past neurosurgical interventions, infections of the central nervous system, normal pressure hydrocephalus, and hereditary diseases of the central nervous system. The disability status of the included subjects was assessed using the Expanded Disability Status Scale (EDSS) (13) based on examinations by experienced neurologists. The study was conducted in accordance with the tenets of the Declaration of Helsinki and approved by the ethics committee of the Ruhr-University of Bochum (REG.Nr: 23-7851).

### 2.2 MR acquisition and post-processing

Images were acquired on a single 1.5 Tesla MRI-Scanner (Aera, Siemens Healthineers, Erlangen, Germany) between September 2018 and March 2022. The protocol consisted of standardized imaging sequences including conventional 3D FLAIR and 3D T1 MPRAGE using a 16-channel head/neck matrix coil. In addition, a 2D axial MDME sequence (repetition time: 6930 ms, echo time 1: 23 ms, echo time 2: 102 ms, inversion time: 29 ms, acquisition matrix: 256 × 146, voxel size: 1 × 1 × 4 mm^3^, and duration: 6 min 34 s) was used and post-processed using the SyMRI^®^ software [version 11.2 for Windows, Synthetic MR, Linkoping, in Sweden; (14, 15)]. Briefly, MDME is a multi-slice, multi-echo, multi-saturation delay acquisition sequence. It allows a simultaneous time efficient quantification of T1 and T2 relaxation times and the proton density. The sequence is based on an interleaved saturation pulse and a Carr-Purcell-Meiboom-Gill acquisition acting on two different slices for the independent estimation of the local B1 field and T1 as well as T2 relaxation times. Synthetic contrasts such as T1-, T2-, PD-w or FLAIR can be generated thereafter using an automated software provided by the vendor (SyMR^®^). For the comparison with conventional contrast-weighted imaging, the sagittal 3D FLAIR sequence (repetition time/echo time/inversion time: 5,000 ms/332 ms/1,800 ms, flip angle 120°, number of excitations: 1, voxel size: 1 × 1 × 1 mm^3^, matrix: 256 × 230, 160 slices, and duration: 4 min 25 s) was used. Both the MDME sequence and the 3D-FLAIR sequence were always performed before administration of contrast agent to avoid the creation of potential interferences.

The post-processing of the MDME-Sequence with the SyMRI software takes < 5 min. Therein choice and generation of synthetic contrasts can be customized and run entirely automated and can be employed within the clinic's internal PACS system without the necessity of exporting images.

### 2.3 Lesion segmentation and MRI data analysis

For the numeric and volumetric assessment of the FLAIR-hyperintense lesions, the Lesion Prediction Algorithm (LPA) included in the Lesion Segmentation Toolbox (LST; version 2.0.15; https://www.statistical-modeling.de/lst.html) was used (16). This deep learning-based algorithm has been validated among several imaging studies and showed satisfactory performance compared to other commonly used methods (17). The LPA employs a binary classifier in the form of a logistic regression model trained on MS patient data. The model produces probability lesion maps and provides numerical outputs for the total number of lesions (TLN) and the total lesion volume (TLV), quantifying the overall brain lesion burden (17).

The 3D FLAIR and the synthetic FLAIR sequences were exported from the PACS system for further analyses and processed on an external stand-alone workstation. To facilitate comparability of the two sequences, 3D FLAIR images were resampled to axial slices with the same spatial resolution as the synthetic images with a slice thickness of 4 mm (named “conventional FLAIR”) before lesion segmentation. To assess spatial accuracy, each individual synthetic image was first linearly registered to its 3D counterpart using FSL's FLIRT registration software, with 12 degrees of freedom. The resulting transformation matrix was then applied to align the lesion map from the synthetic image into the 3D FLAIR image space. The registered lesion maps were subsequently used to calculate the Sørensen–Dice Coefficient (SDC) between the synthetic and conventional segmented areas. This coefficient quantifies the percentage of spatial overlap between two segmented areas, ranging from 0 (no similarity) to 1 (complete similarity) between the two segmentations. The SDC was calculated using the formula (2 × intersection area)/sum of synthetic and conventional lesion areas.

All images, along with their segmented areas, were registered onto a common brain template to further enhance visualization of lesion distribution and to identify the existence of lesion patterns within certain brain regions. We used a FLAIR template [GG-FLAIR-366, available at http://brainder.org; (18)], to which the individual conventional FLAIR images were linearly and non-linearly aligned using FSL's FLIRT and FNIRT registration software tools. The resulting warping field was then applied to the segmentation masks of both the conventional FLAIR image and the previously registered synthetic image. Average lesion maps were calculated for both the synthetic and conventional FLAIR segmentations. For visualization purpose, only lesion areas that appeared in >5% of participants are shown to minimize false-positive lesion segmentations.

### 2.4 Statistical analysis

Statistical analysis and visualizations were conducted using SPSS version 28 (IBM Corporation, Armonk, NY, USA). Results were considered significant at *p* < 0.05. All parameters were tested for normality using the Shapiro–Wilk test. Since both TLV and TLN data were not normally distributed, the non-parametric Wilcoxon signed-rank test for paired data was used to determine significant difference between the LPA segmentation results in the synthetic vs. conventional images.

To assess possible differences between the MS subgroups, a Mann-Whitney U test was performed between the TLV values of the relapsing-remitting MS subgroup (RRMS) and the progressive MS subgroup (PMS, including patients with primary and secondary progressive MS). In addition, a box plot was created for visualization.

Bland–Altman plots were generated for visualization of the spread in TLV and TLN data, providing a comprehensive statistical presentation. In addition, intraclass correlation coefficient (ICC) was calculated to assess the agreement of the two measurements of these two variables. According to statistical conventions, ICC ratings of 0.40, 0.40–0.59, 0.60–0.74, and 0.75–1.0 were considered to represent weak, fair, good, or excellent agreement, respectively (19). The ICC was calculated using a two-way-mixed-(effects) model that measured absolute agreement.

To assess the spatial accuracy, the SDC was calculated for the registered lesion maps. A Spearman rank correlation test was performed to investigate the correlation between the calculated SDC values and the TLV measured with conventional FLAIR. Additionally, an independent two-group Mann–Whitney U test was used to evaluate the differences in SDC between the groups with a medium-to-high TLV (>5 mL) and a low TLV (< 5 mL).

For effect size evaluation, the rank biserial correlation (r) was calculated using the formula $r\;=z/\sqrt{N}$, where z is the standardized test statistic, and *N* is the number of individual participants.

Furthermore, Spearman rank correlation tests were employed to examine the correlation between the different lesion volumes, lesion number and the EDSS, as well as the lesion loads and the disease duration.

## 3 Results

The final patient cohort, selected according to the inclusion and exclusion criteria comprised 156 patients with all major subtypes of MS (Table 1). The mean disease duration of MS subjects was 15.4 ± 11.2 years with a median EDSS of 4. Detailed demographic characteristics of the study population are presented in Table 1.

**Table 1:** Demographics of the study population.

**Characteristics**	**Findings**
Participants (n)	156
Sex (female/male)	89/67
Age (years)	49.4 ± 13.5
MS subtype (RR/PP/SP); (n)	70/22/64
EDSS score (median (IQR))	4 (2)
Disease duration (years)	15.4 ± 11.2

Data are shown as mean±SD unless otherwise specified.

EDSS, Expanded Disability Status Scale; IQR, interquartile range; PP, primary-progressive; RR, relapsing-remitting; SD, standard deviation; SP, secondary-progressive.

**Figure 1:**
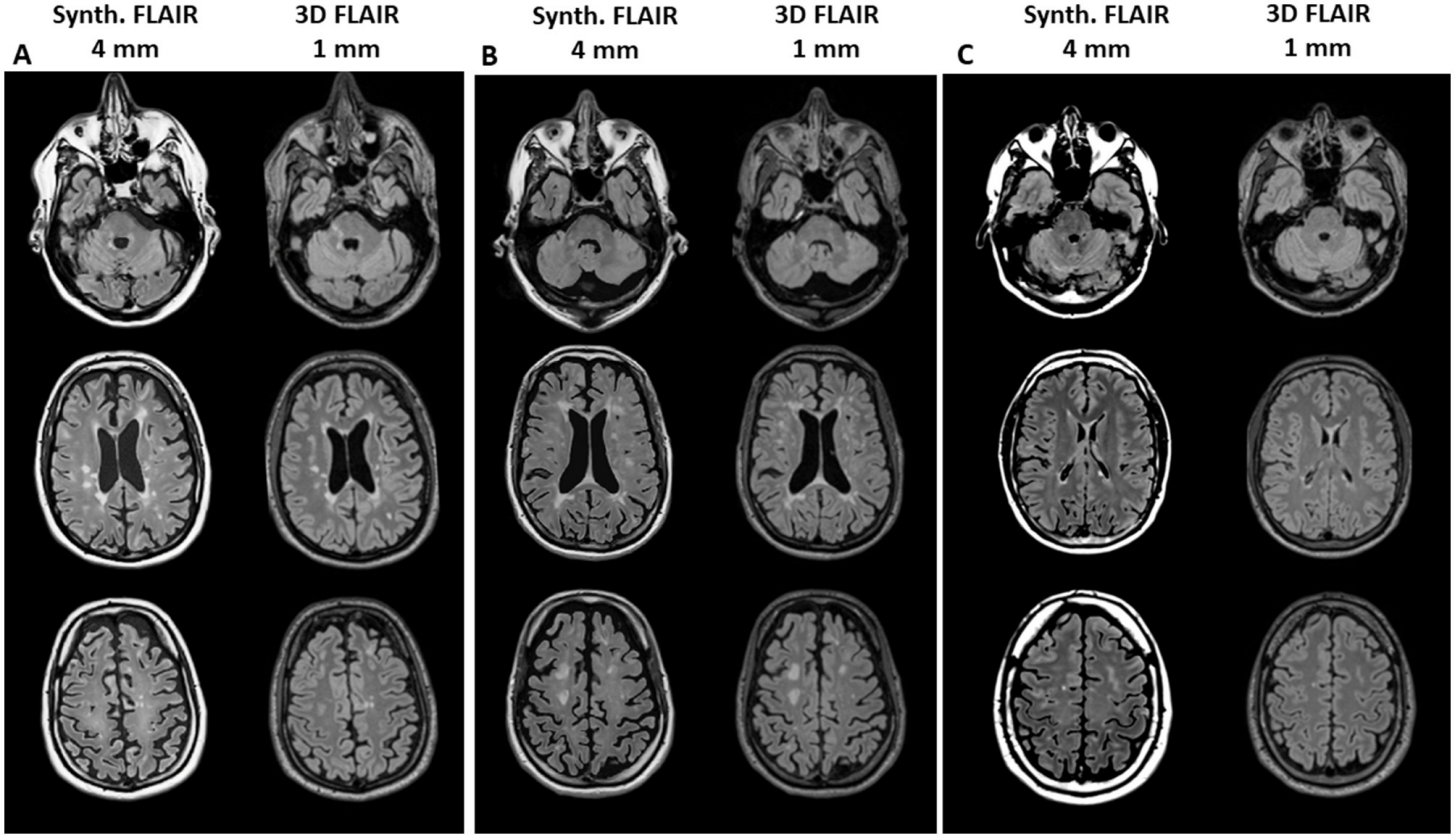
Example images of three patients with high lesion volume [**(A)** male, age 50 years, SPMS, TLV 28 ml and **(B)** male, age 38 years, RRMS, TLV 27 ml] or low lesion volume [**(C)** male, age 30 years, RRMS, TLV 0.5 ml] for visual comparison between synthetic FLAIR images (with 4 mm slice thickness, left column in each block) and the original 3D FLAIR images (1 mm slice thickness, right column). The upper row shows infratentorial sections, the middle row shows ventricular sections, and the lower row shows supraventricular sections.

Patients in the advanced disease stages, such as those with secondary progressive MS (SPMS), exhibited notably higher lesion loads than others. Figure 1 shows exemplary images of three patients with high or low lesion load comparing the visual impression between synthetic FLAIR images and the original 3D FLAIR images. Considering the different slice thicknesses, the synthetic and high-resolution conventional FLAIR sequences show similar visibility of MS lesions on the ventricular and supratentorial slices. Lesions in the infratentorial area are more clearly identifiable on the 3D FLAIR sequences.

**Figure 2:**
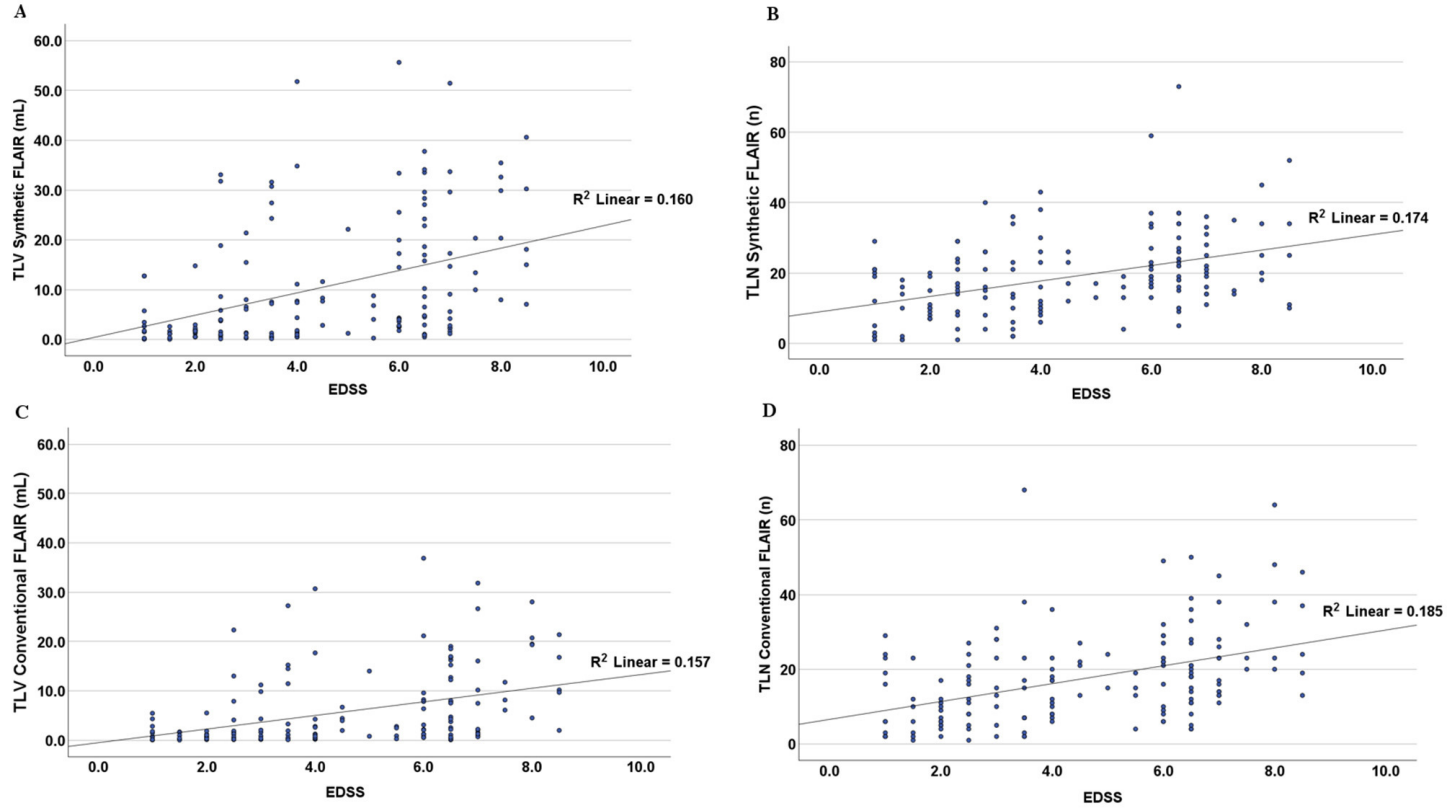
Scatter plots showing the relationship between the Expanded Disability Status Scale (EDSS) and total lesion volume (TLV, in **A, C**) and total lesion number (TLN, in **B, D**) as measured in synthetic and conventional FLAIR sequences. Linear regression lines and the corresponding R^2^ values were added to the plots. The graphs contain segmented numerical and volume data from all singular cases included in this study.

In our statistical analyses, a significant positive correlation was found between disease duration and the total lesion load in the synthetic and conventional FLAIR sequences (rho of 0.44 and *p* < 0.001 in both instances). Furthermore, correlation analysis between TLV and EDSS yielded a rho of 0.46 for TLV Synthetic FLAIR and a rho of 0.45 for TLV conventional FLAIR (*p* < 0.001). A similar correlation was observed between total lesion number (TLN) and EDSS, with rho values of 0.43 for synthetic FLAIR and 0.46 for conventional FLAIR (*p* < 0.001; Figure 2).

**Figure 3:**
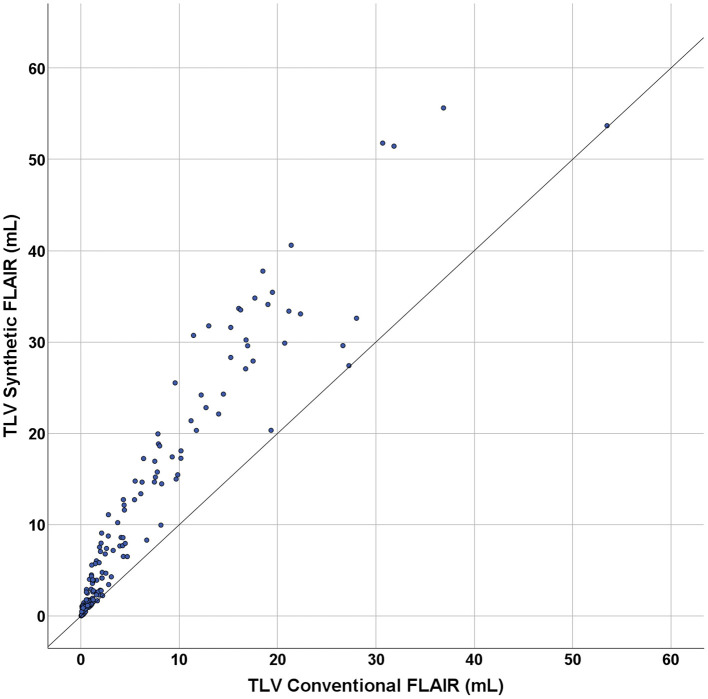
Scatterplot showing the plotting of the total lesion volume (TLV) conventional FLAIR (in mL) against the total lesion volume of the synthetic FLAIR (in mL). The diagonal line shows the optimal hypothetical conversion between synthetic and conventional images of the total lesion volumes. The graph contains the segmented volume data from all singular cases included in this study.

**Figure 4:**
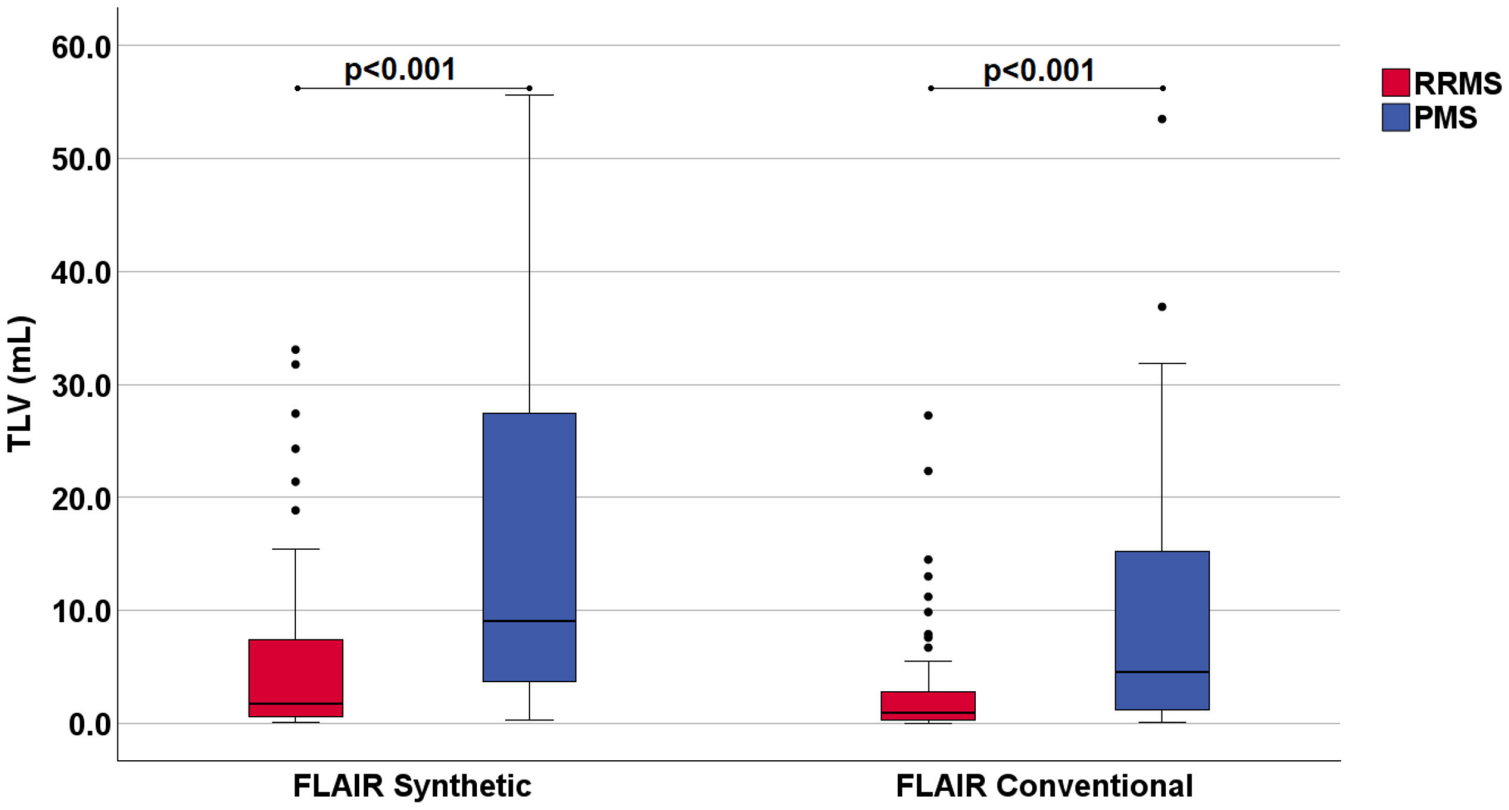
Boxplots showing the measured total lesion volume (TLV) in milliliters (mL) in synthetic and conventional FLAIR images. The data is categorized into two subgroups of Multiple Sclerosis (MS): Relapsing-Remitting MS (RRMS) and Progressive MS (PMS). Patients with Primary and Secondary Progressive MS were included into the PMS subgroup. The *p*-values resulting from the Mann-Whitney-U test, which demonstrated a statistically significant difference between the TLV of the individual groups, have been included at the top of the graph. The boxplot contains the segmented volume data from all singular cases included in this study.

**Figure 5:**
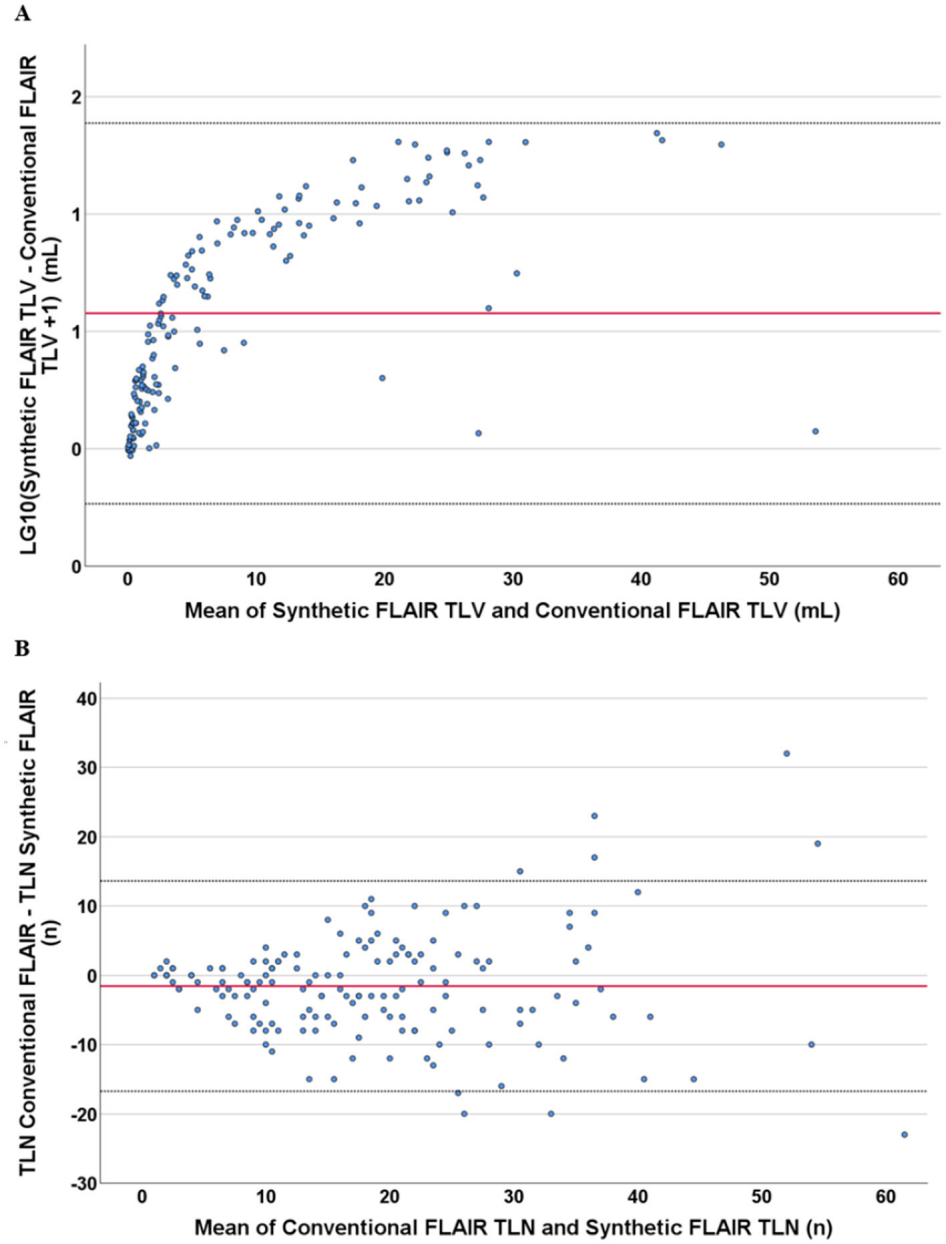
Bland–Altman plots showing the relation of the differences with the measured mean values, both in respect to the total lesion volume and number measurement in synthetic FLAIR and conventional FLAIR sequences. Both graphs contain data from all singular cases included in this study. According to Bland–Altman analysis, the red line shows the mean, and the dotted black line shows the 1.96-fold standard deviation. **(A)** shows the logarithmized difference of the measured volume of lesions in synthetic and conventional sequences against the mean values of the same volumetric measurement. The logarithmization was performed because of the non-Gaussian distribution of the measured differences in volume. **(B)** shows the difference of the measured number of lesions in synthetic and conventional sequences against the mean values of the same numerical measurement.

The TLV measurements from synthetic images were consistently higher than those obtained from conventional images at both the individual and group levels (Figures 3, 4, Table 2). Slightly more segmented lesions were identified in the synthetic images (Table 2). The Wilcoxon signed-rank tests showed that both volume and number of lesions differed significantly between synthetic and conventional FLAIR imaging (*p* < 0.001 for TLV, and *p* < 0.003 for TLN). A significant difference between the TLV values of the RRMS and PMS subgroups was found for both the synthetic and conventional TLVs (*p* < 0.001, in both instances). Boxplots indicated lower variability in TLV values within the RRMS subgroup compared to the PMS subgroup, and lower variability in conventional TLV values compared to synthetic TLV values (Figure 4). The Bland-Altman analysis showed that cases with a lower overall TLV exhibited better agreement between synthetic and conventional lesion volumes than cases with a higher overall TLV (Figure 5A). Regarding the TLN, slight increases in dispersion and numerical difference between conventional and synthetic FLAIR were observed as lesion number increases (Figure 5B). In both plots, nearly all cases fell within the confidence interval, suggesting reliable measurements based on Bland-Altman analysis standards (20). Due to the non-gaussian distribution of the lesion volume differences, a logarithmic transformation was applied. The intraclass correlation coefficient (ICC) for TLV was 0.885, and for TLN it was 0.89, both indicating excellent agreement (19).

**Table 2:** Lesion segmentation results [total lesion volume (ml), total lesion number (n)] based on synthetic FLAIR and conventional FLAIR images.

	**TLV Syn. FLAIR (mL)**	**TLV Conv. FLAIR (mL)**	***p*-value**	**TLN Syn. FLAIR (n)**	**TLN Conv. FLAIR (n)**	***p*-value**
Mean	11.0	6.1	< 0.001	19.5	17.9	< 0.003
SD	12.8	8.5	–	12.1	12.5	–
Range	0.4–55.6	0.2–53.5	–	1–73	1–68	–

The conventional FLAIR images are based on isotropic 3D FLAIR reformatted to 4-mm-slice thickness. The significance (*p*-value) of group difference is assessed by the Wilcoxon signed-rank test.

syn., synthetic; conv., conventional; SD, standard deviation; TLN, total lesion number; TLV, total lesion volume.

The spatial similarity assessment showed a mean SDC of 0.47 ± 0.15. The Spearman test showed a significant positive correlation between the SDC and the overall TLV from conventional FLAIR imaging (rho = 0.57; *p* < 0.001). A Mann–Whitney U test showed that cases with higher lesion loads (>5 mL) had significantly higher SDC values than those with lower loads (< 5 mL; *p* < 0.001), indicating greater similarity between synthetic and conventional FLAIR at higher lesion loads than at lower lesion loads. Specifically, the mean SDC for cases with TLV < 5 mL was 0.42 ± 0.14, compared to 0.57 ± 0.11 for cases with TLV >5 mL. This meant that lesion maps with high lesion load showed higher similarity between synthetic and conventional FLAIR than lesion maps with low lesion load. The rank biserial correlation coefficient for this correlation was 0.52, indicating a strong effect (21).

**Figure 6:**
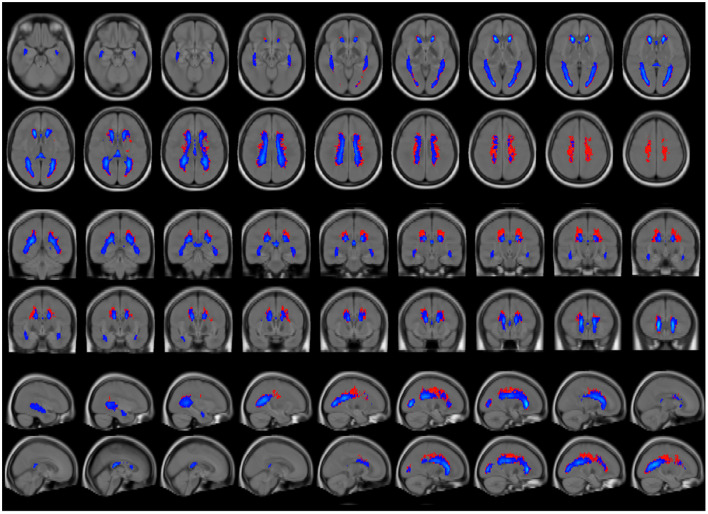
Average lesion maps of the entire patient group that were generated for segmentation results based on synthetic and conventional FLAIR images. The synthetic lesion segmentations are depicted in red, and the conventional FLAIR lesion segmentations are represented in blue. To enhance clarity in visualization, only lesion areas present in >5% of the participants are displayed, thus reducing the display of excessive false-positive lesion segmentations. The lesion maps are overlayed on a T1w brain template.

To directly compare lesion locations within the brain, average lesion maps from synthetic and conventional FLAIR images were superimposed (Figure 6). Periventricular areas, as shown on the average lesion maps (Figure 6). Synthetic FLAIR images presented larger lesion areas, predominantly located at the superior corona radiata, and extending to the outer borders of the supratentorial regions, as opposed to conventional FLAIR images.

## 4 Discussion

This study focused on the evaluation of lesion quantification that is crucial for the radiological assessment of MS. The aim was to improve the knowledge of synthetic FLAIR imaging and determine whether the technology is a viable option for future clinical imaging in MS. Prior studies have suggested an inferiority in the quality of synthetic FLAIR images compared to conventional FLAIR images (22), citing increased artifacts such as flow artifacts, white noise, and granulated artifacts near cerebrospinal fluid surfaces (9, 12, 22, 23).

In contrast to the study of Fujita et al., which had similar characteristics (22), we found a significant difference in the lesion count and volume between synthetic and conventional FLAIR sequences, possibly attributable to the differences in slice thicknesses of synthetic images between our study (4 mm) and that of Fujita et al. (1.3 mm). This discrepancy implies that common synthetic FLAIR artifacts may be driven by partial volume effects, which, by its nature, increases with greater slice thickness. It is noteworthy that Mann-Whitney U test for TLV values between the RRMS and the PMS subgroup yielded similarly low *p*-values in both cases, despite greater variability within the synthetic segmented data compared to conventional FLAIR in each group. This finding highlights that lesion segmentation across both sequences yields comparable relevant clinical information. Supporting this is the observed relationship between TLV/TLN and the EDSS. The Spearman tests indicate that lesion segmentation using synthetic FLAIR successfully replicates the well-established correlation between EDSS and MS lesion burden (24). This suggests that synthetic FLAIR lesion segmentation is reliable, further strengthening its potential applicability in clinical practice. In the Bland-Altman plot analysis, nearly all cases fell within the confidence interval for both TLV and TLN analysis, suggesting reliable measurements according to the Bland-Altman analysis standards (20). The ICC (values) were also excellent, indicating a strong agreement between measurements obtained from synthetic and conventional FLAIR (19).

The study's evaluation of spatial accuracy yielded moderately accurate SDC results, aligning closely with findings by Ribaldi et al. (17) who observed similar LST-LPA performance on FLAIR sequences, though they compared results with manual segmentation. Like our study, Ribaldi et al. reported higher SDC values in patients with greater lesion burden. This pattern likely reflects, as Rainai et al. (25) suggested, that higher lesion loads leave less space for false positives and false negatives, resulting in more accurate segmentations and higher SDC values. In addition, if the volume of the lesion areas increases within the limited space of a patient's brain, the likelihood of overlap in the **two** segmentations also increases with it, leading to a consequently higher SDC. Nevertheless, SDC cannot be regarded as a precise metric for segmentation accuracy, and its limitations should be acknowledged.

Another noteworthy finding relates to the observed differences between the average lesion maps shown in Figure 6. Most deviations are located in periventricular areas with a supraventricular emphasis, which could be attributed to an increased accumulation of partial volume effects in these regions. This interpretation is supported by the larger TLV differences observed in patients with higher overall lesion burdens and the fact that such patients often exhibit ventricular enlargement, a common feature associated with increased lesion burden in MS (26). The resulting expansion of the brain's inner surface area in these patients may also lead to a greater number of voxels, containing a mix of cerebrospinal fluid and tissue signals, possibly increasing the potential for possible artifact generation.

In summary, the advantages of synthetic imaging are apparent. Agreement between conventional FLAIR in lesion quantification was excellent (19), and the spatial accuracy was adequate for radiological/clinical assessment. In patients with lower lesion burden, conventional and synthetic FLAIR imaging yielded similarly small disparities. Overall, the singular MDME sequence stands out as a swift MRI sequence with its comparatively short acquisition time suited to generate a set of image series with contrast needed for a standardized MS protocol. This attribute proves notably beneficial in the context of MS diagnostics, as the disease necessitates frequent cranial scans for both diagnostics and monitoring. Furthermore, another advantage lies in its potential for standardization across clinical care settings, as the sequence parameters are predefined, eliminating the need for intricate adjustments. Particularly for radiological facilities without access to 3D FLAIR, this technology could provide an opportunity for better standardization. Another benefit could be the consistency in slice positioning between synthetic sequences, resulting from the use of the same source dataset, thus reducing common sources of error in axial 2D images.

However, some limitations must be considered when interpreting the present results. This study did not include manual segmentation as the gold standard, although LST segmentations have been widely used and validated for the segmentation of MS lesions. Still, our focus was comparing the performance of the LST software tool between conventional FLAIR and synthetic FLAIR imaging, whereby the accuracy of LST using conventional FLAIR imaging has widely been shown in previous studies and was thus regarded as an established reference (17). Consequently, we have not done manual lesion delineation and counting and we cannot provide information about the accuracy of lesion detection in different brain regions. In previous studies, overall good agreement between lesion count using conventional and synthetic FLAIR imaging has been found, but less agreement in infratentorial regions than in deep white matter or periventricular WM (11, 12). Still, LST only provides the total lesion count and volumes without differentiating between different brain regions, which has to be acknowledged as a limitation. Furthermore, the monocentric nature of this study and the use of only one scanner limits the generalizability across different equipment, as it did not comprise the whole range of contrasts emerging from different scanners. Thus, there is a need for large multicenter studies to further investigate MS lesion segmentation in synthetic FLAIR sequences.

It is also important to acknowledge that synthetic FLAIR images exhibit a reduced spatial resolution in comparison with conventional 3D-FLAIR images. The trade-off, however, could be justified by the fact that the MDME-sequence offers not only an alternative set of contrasts but also can generate a variety of quantitative parameters, namely volumes and segmentation maps of total brain, gray and white matter, and advanced quantitative measures such as maps of myelin, proton density and T1 and T2 relaxation times. As posited by Cao et al. (27) MS patients exhibited significant alterations in global and regional brain volumetry and relaxometry as measured by the MDME sequence. Brain atrophy, for instance, is an important factor in the evaluation of subsequent disability and disease severity. These quantitative parameters, therefore, have the potential to serve as an auxiliary tool for monitoring and diagnosing MS. The automatic calculation of brain volume (brain parenchymal fraction), whose reliability has recently demonstrated in comparison with other established methods (28), probably has the greatest practical significance for clinical diagnostics, as the examination of brain atrophy is known to be an important prognostic marker for the progression of MS (29). High resolution 3D synthetic MRI (at higher acquisition times) will probably become an alternative to 2D MDME imaging, with the recently developed 3D- QALAS sequence (30). Still to date, the 3D-QALAS sequence is not yet available for clinical use by all MRI vendors. Finally, longitudinal studies should examine the sensitivity of synthetic sequences in terms of assessing disease-related temporal dynamics such as detecting active T2 lesions or dissemination in time. Although Synthetic MR has made significantly efforts to reduce artifacts in the transition from SyMRI^®^ version 11 to 12, artifact-related challenges remain a primary limitation of this technique and warrant further improvement. Deep learning-based approaches, such as conditional generative adversarial networks trained with a pixel-wise translation approach could offer an effective solution for improving the quality of synthetic FLAIR images in the context of MS lesion assessment (22). Aymerich et al. found that trained radiologists could still interpret synthetic images despite artifacts, supporting its clinical use (31). In addition, findings from Fujita et al. and our study suggests that reducing the slice thickness may reduce artifacts, potentially improving synthetic FLAIR's accuracy.

## 5 Conclusion

The findings of this study indicate that synthetically generated FLAIR sequences offer a promising, reliable, accurate, and efficient option for future MS imaging. In particular, synthetic imaging's reduced acquisition time presents a practical advantage. Our results demonstrate that synthetic FLAIR sequences can effectively assess MS lesions, supported by an excellent measurement agreement and satisfactory spatial accuracy compared to conventional FLAIR. Importantly, synthetic FLAIR sequences show strong accuracy specifically in patients with lower lesion burden, highlighting the usability in the early stages of MS.

## Data Availability

The datasets presented in this article are not readily available because patients' raw MRI and clinical data cannot be made available due to data protection regulations. Other data can be shared on reasonable request to the corresponding author. Requests to access the datasets should be directed to Carsten Lukas, carsten.lukas@ruhr-uni-bochum.de.
